# Materia Medica and Therapeutics, with Especial Reference to the Clinical Application of Drugs

**Published:** 1891-06

**Authors:** 


Materia Medica and Therapeutics, with Especial Reference to the Clinical Application of Drugs. By John V. Shoemaker, A. M., M. D., Professor of Materia Medica, etc., in the Medico-Chirurgical College, of Philadelphia. Vol. II being an independent volume upon drugs. Philadelphia (1231 Filbert Street), and London, 1891. Price, cloth, $3.50 net; sheep, $4.50 net.

This work is devoted to materia medica from the stand-point of the Old School or Regular School of medicine. It contains all the remedies and measures of the old school arranged in alphabetical order. The information is full and yet concise. It is an excellent exposition of the sphere of drugs, but, of course, is not so useful to a physician of the Homœopathic School, except as a book of reference to throw light upon the origin of some of the remedies of our own school. Still it is a most excellent book, and is brought up to date in its information. It contains about 1,000 pages, and is well printed.

